# The role of congruence: a response surface analysis of family-school alignment in predicting creative thinking

**DOI:** 10.3389/fpsyg.2026.1926749

**Published:** 2026-09-23

**Authors:** Osman Tat

**Affiliations:** Educational Sciences, Faculty of Education, Van Yuzuncu Yil University, Van, Türkiye

**Keywords:** congruence, creative activity, creative thinking, cubic polynomial regression, openness, person-environment fit, response surface analysis

## Abstract

**Background/objectives:**

Despite widespread acknowledgment that school and home environments must be aligned to support cognitive development, the question of how their congruence or incongruence predicts creativity has received little empirical attention.

**Methods:**

Drawing on data from the PISA 2022 Creative Thinking Assessment, this study employed multilevel cubic polynomial regression and response surface analysis (RSA), with students nested within countries to examine how three congruence pairs: (1) environmental support, (2) behavioral engagement and (3) value orientation predict creative thinking performance controlling for mathematics, science and reading achievement and socioeconomic status.

**Results:**

Results revealed that aligned increases along the line of congruence were associated with higher creative thinking in the environmental support and value orientation, but with lower creative thinking in the behavioral engagement model where uniformly high participation in both activity domains was associated with the poorest outcomes. The quadratic congruence parameter was negative and significant, indicating diminishing rather than accelerating returns to congruence. Regarding compensatory mechanisms, the family environment partially buffered school deficiencies, an excess of out-of-school over in-school activity was associated with lower scores than the reverse imbalance and student openness to experience substantially compensated for a low school openness culture, whereas the reverse configurations conferred no comparable advantage. Across all three models the congruence terms accounted for considerably more of the within-country than the between-country variance. The full models explained 55% of the within-country and 78–80% of the between-country variance; once achievement and socioeconomic status were controlled, the unique contribution of the congruence terms ranged from 3.0 to 6.7%.

**Conclusion:**

These findings support a dynamic, configural view of creativity as an emergent outcome of multi-contextual fit and underscore the need for integrated intervention designs. The asymmetric compensatory patterns revealed demonstrate that creativity support systems are not interchangeable across contexts and that targeted, context-sensitive strategies are necessary to foster creative potential. Because the congruence effects operate at the level of the individual student rather than the education system, and are modest in magnitude, they identify a direction for such strategies rather than a basis for redesigning provision around them.

## 1 Introduction

Csikszentmihalyi (2014) argued that creativity which has been recognized as one of the most critical skills of the 21st century (Barbot and Kaufman, 2025) would serve as the sole agent in solving our planet’s long-standing problems of coexistence on an increasingly interdependent globe. Given today’s pressing challenges and the unpredictable complex problems of the future, greater investment in creativity, humanity’s most important tool for struggle, seems inevitable (Pugsley and Acar, 2020). Indeed, education systems have begun placing creativity at the center of educational reforms to cultivate individuals capable of coping with current and future challenges in both personal and professional contexts and providing the innovation needed across multiple domains (Gelmez Burakgazi and Reiss, 2025; Kraus et al., 2026). The OECD’s inclusion of creativity as an innovation area in large-scale international assessments such as PISA is one of the most concrete indicators of this trend (Hoffmann et al., 2025; Kraus et al., 2026).

Creativity, which is deemed so important, has been approached by many researchers not as a static personal trait but as a dynamic phenomenon shaped by environmental conditions. For instance, Walia (2019) views creativity as continuous actions that begin with the emergence of an imbalance in the environment and go beyond conventional patterns of thought. Mockros and Csikszentmihalyi (1999), on the other hand, stated that creativity is not a property of individuals but rather of social systems that make judgments about individuals. Similarly, according to the Componential Model of Creativity which is one of the most influential theories in the creativity literature, creativity is not merely a static individual trait but a dynamic phenomenon shaped by environmental conditions (Amabile, 1983; Amabile and Pratt, 2016; Hennessey and Amabile, 2010). In her theory, Amabile (1983) explains how intrapersonal factors support progress toward a goal and what role socio-environmental factors play in this process (Amabile, 1983; Ginns et al., 2021). The componential theory includes three intrapersonal components affecting creativity: domain-relevant skills (expertise in the relevant domain or domains), creativity-relevant processes (cognitive and personality processes that facilitate novel thinking) and task motivation. The component outside the individual is the surrounding environment, particularly the social environment (Amabile, 1983; Amabile and Pillemer, 2012). The author explains how these four components shape the mechanism of creative thinking as follows: Domain-relevant skills can be defined as the area of expertise, technical knowledge, or ability in which the individual will perform the creative act. A deficiency in this component, which forms the foundation of the creative process, can render creativity impossible (Amabile and Pillemer, 2012; Amabile and Pratt, 2016). The creativity-relevant cognitive processes component encompasses cognitive style, heuristics for generating novel ideas and working style. Task motivation includes attitudes toward the task and perceptions of one’s own motivation. The final component, the social environment, is the external component that can nourish or impede any of the previous three components (Amabile and Pillemer, 2012).

The social environment component in Amabile's (1983) Componential Model of Creativity can be considered as a microsystem in Bronfenbrenner's (1979) ecological systems theory, which provides a powerful theoretical framework for understanding the social context of development. According to the theory, the microsystem is defined as the family, school, peer, or work environments with which the individual directly interacts. The mesosystem is the second layer encompassing the relationships and interactions between two or more microsystems. According to this model, microsystems can foster creativity by supporting autonomy, keeping curiosity alive and transforming mistakes into learning opportunities, thereby enhancing students’ self-efficacy and intrinsic motivation. The power of mesosystems emerges not merely from the co-presence of microsystems but from the consistency between them. In the context of creativity, the collaboration or conflict between students’ home and school environments (parents and teachers) is known to be vital (Gelmez Burakgazi and Reiss, 2025). Similarly, creativity is not immune to the shaping influence of cultures, not just environments. Creative thinking is directly influenced by culture both inside and outside school, as well as by parent and teacher culture and student culture (Kyritsi and Davis, 2021; Niu and Kaufman, 2013; Runco et al., 2017). Finally, school environments that support students with a culture open to creativity and integrate creative activities and tasks into the curriculum can achieve highly effective results when combined with participation in extracurricular creative activities (Barbot and Kaufman, 2025).

The creative thinking literature hosts numerous studies examining creative school environments (Huang et al., 2021; Runco et al., 2017), school culture or culture of openness to creativity (Gelmez Burakgazi and Reiss, 2025; Ginns et al., 2021; Hoffmann et al., 2025; Karwowski, 2011; Zibenberg and Da'as, 2026) and the effect of the intensity of creative activities at school on creativity (Chaiyarat, 2024; Rodríguez et al., 2019; Weng et al., 2023). Similarly, the effects of creative home environment (Jankowska and Karwowski, 2019; Pugsley and Acar, 2020; Routledge et al., 2008), student openness to creativity (Agnoli et al., 2018; Karwowski and Lebuda, 2016; Oleynick et al., 2014) and the intensity of out-of-school creative activities (Milgram and Hong, 1999; Runco et al., 2017) have been examined separately in numerous scientific studies. However, no study has examined the effect of congruence between school and out-of-school environments on creativity by treating Amabile’s social environment component as Bronfenbrenner’s mesosystem concept and presenting empirical results. It is widely accepted that school and home environments (learning and family, etc.) must be aligned in supporting many cognitive or affective characteristics. However, there are no scientific findings regarding the optimal level of congruence, whether congruence behaves the same way across all cognitive levels, or whether any component can compensate for the deficiency of another in cases of incongruence.

## 2 Theoretical framework

### 2.1 Creative school and home environment

Creative school climate is a school environment that includes interpersonal trust, support for the emergence of new ideas and dynamism that triggers willingness to take risks (Bereczki and Kárpáti, 2018; Hoffmann et al., 2025; Karwowski, 2011). A school environment where psychological safety is established, as a shared feeling that making mistakes and failing are acceptable, can increase students’ intrinsic motivation and thus their creativity by providing access to developmental opportunities (Amabile and Pratt, 2016; Ginns et al., 2021; Hoffmann et al., 2025; Rogers, 1954). Rogers (1954) argued that constructive creativity can emerge when an environment offers psychological freedom alongside psychological safety. The literature emphasizes that teachers witnessing colleague innovation and general instructional collaboration are school-level facilitators that support creativity (Adams et al., 2025; Bereczki and Kárpáti, 2018; Hoffmann et al., 2025; Huang et al., 2021). Open-ended parameters provided by teachers not only enable students to acquire domain-specific technical skills but can also encourage original creations by breaking mental patterns (Kukkonen and Bolden, 2022). Moreover, it is known that not only the teacher dimension but also school management’s recognition and support of creative teaching feeds teachers’ self-efficacy in developing long-term creative projects (Fischer, 2020). However, as noted by Amabile (1983, 1996) and Bereczki and Kárpáti (2018), school deficiencies such as inflexible programs, intense competition and excessive dependence on extrinsic rewards are counted among the most important barriers to creativity (Amabile, 1983; Amabile and Pratt, 2016; Bereczki and Kárpáti, 2018; Cullingford, 2007; Moore, 2007).

Just like the school environment, parents displaying positive attitudes toward creativity and providing creative environments at home directly and positively affect their children’s creativity (Jankowska and Gralewski, 2022; Jankowska and Karwowski, 2019; Pugsley and Acar, 2020; van der Zanden et al., 2020). In this process, a complex family structure that balances integration and differentiation (Pugsley and Acar, 2020) along with psychological safety and freedom form the constructive foundation for creativity. The intellectual climate established at home, the prominence of parental autonomy and the consequent shaping of children’s task motivation (Amabile, 1983; Amabile and Pratt, 2016) reveal the multidimensional effect of family on children’s creativity. However, it can be said that families’ lack of social and economic capital (Csikszentmihalyi, 1999; Jankowska and Karwowski, 2019; van der Zanden et al., 2020) and their excessive performance orientation negatively affect children’s creative thinking (Jankowska and Karwowski, 2019).

The literature indicates that the likelihood of creativity emerging reaches its highest levels when both challenging and supportive school and home environments coexist (Gelmez Burakgazi and Reiss, 2025; Runco, 2006). This is because creative children need guidance both at school and at home and incongruence between these two environments is known to cause a creativity gap (Runco et al., 2017).

### 2.2 Creative activities at school and home settings

Students’ participation in creative activities differs markedly between school and home contexts. Runco et al. (2017), in their study with 254 Turkish university students, found that students engaged in significantly more creative activities outside school compared to inside school and correlations between the two contexts were quite low (highest r = 0.15). Recent research indicates that the ways school and home environments support creativity are more complex than previously thought. In the school context, while some studies have unexpectedly found negative relationships between participation in creative activities and creative thinking (Hernández-Ramos and Araya, 2025), other research emphasizes that this may be related more to the quality of activities than their mere presence. Teachers’ limited understanding of creativity (Newton and Newton, 2010), standardized and assessment-oriented classroom climates (Can and Bart, 2026) and the importance of creativity-supportive teacher behaviors and openness personality traits (Du et al., 2019; Adams et al., 2025) demonstrate that creativity in school settings is largely shaped by pedagogical and psychological processes. Similarly, the fact that stress reduces creative tendencies while openness can weaken this negative effect (Wang et al., 2026), the positive contributions of creative physical and social learning environments (Hyndman and Mahony, 2018) and the decisive role of intrinsic motivation (Wu, 2026) reveal that the school’s effect on creativity has a multidimensional structure.

Findings regarding the home environment present a similar picture. Research shows that parental support affects creativity not directly but mostly through mechanisms such as autonomy and intrinsic motivation. Additionally, openness personality trait and intrinsic motivation have been found to be strongly associated with creative achievement, while extrinsic motivation is linked to weaker or negative outcomes (Kaufman et al., 2015; Prabhu et al., 2008). Taken together, these studies demonstrate that creativity is nourished not merely by the number of activity opportunities but by the autonomy support, motivational climate, personality traits and quality of social interactions provided in school and home environments.

### 2.3 Openness to experience and the school’s culture of openness

Students’ openness to creativity and creative self-efficacy beliefs are among the most studied factors at the individual level in the literature (Agnoli et al., 2018; Du et al., 2019; Ginns et al., 2021; Karwowski and Lebuda, 2016; Oleynick et al., 2014). The emergence and maintenance of creativity exhibits a multi-level structure based on the dynamic interaction between individual characteristics and environmental factors. At the individual level, students’ openness to novelty and experience personality traits along with their intellectual curiosity directly shape their artistic and scientific creative achievements (Kaufman et al., 2015). However, these individual tendencies are closely related to classroom dynamics; indeed, teachers’ creativity-fostering behaviors nourish creative achievement by increasing students’ intrinsic motivation, but this positive process finds strong resonance particularly among students who are already open to novelty by nature (Du et al., 2019). At the institutional level, a school culture that supports innovation, risk-taking and open communication facilitates teachers’ implementation of creative pedagogies in the classroom and this institutional effect becomes more pronounced with teachers’ professional seniority (Zibenberg and Da'as, 2026). Similarly, teachers who are open to innovation as a personality trait have higher levels of their own intrinsic creativity and this potential is directly reflected in innovative teaching practices in the classroom (Adams et al., 2025). In conclusion, the literature reveals that creative processes constitute a holistic mechanism nourished simultaneously by both the individual ‘openness’ tendencies of students and teachers and the institutional ‘openness’ culture of the school environment.

### 2.4 The present study

This study aimed to reveal how creative cognitive ability is predicted by response surface analysis based on: the congruence between creative school environment and creative home environment, the congruence between creative activities at school and out-of-school activities and finally the congruence between the school’s culture of openness and the student’s openness to experience. Response surface analysis was used to examine both congruence and incongruence patterns across these three pairs.

The relationships examined in this study may not remain constant across all levels of the predictors. Creativity-supportive conditions may show diminishing returns at higher levels, while some effects may emerge only after a certain level is reached (Rogers, 1954; Amabile and Pratt, 2016; Jauk et al., 2013). A quadratic response surface assumes constant curvature along a given line, whereas a cubic specification allows the rate and curvature of the relationship to change across predictor levels. Therefore, cubic RSA was used to represent possible level-dependent patterns in the correspondence between the paired variables.

Previous studies show that both school and home environments are important for students’ creativity (Rogers, 1954; Pugsley and Acar, 2020; Hoffmann et al., 2025). Creativity may be stronger when both environments provide supportive conditions, while inconsistency between these contexts may create a creativity gap (Runco, 2006; Runco et al., 2017). These findings suggest that examining school and home environments only as separate predictors may overlook the effect of their joint configuration. Therefore, examining the correspondence between these two environments may provide a better understanding of students’ creative thinking.

- RQ1. How is creative thinking related to the correspondence between the creative environments students experience at home and at school?

   ○  H1a. Along the line of congruence, creative thinking increases as family and school creative environments increase together (u1 > 0).
   ○  H1b. Along the line of incongruence, creative thinking is higher when the family creative environment exceeds the school creative environment than in the reverse direction (v1 > 0).

Previous studies suggest that creative activity participation inside and outside school may not contribute to creativity in the same way (Runco et al., 2017; Hernández-Ramos and Araya, 2025). Therefore, examining the correspondence between these two activity contexts may provide a better understanding of their relation to creative thinking.

- RQ2. How is creative thinking related to the correspondence between participation in creative activities inside and outside school?

   ○  H2a. Along the line of congruence, creative thinking increases as participation in creative activities inside and outside school increases together (u1 > 0).
   ○  H2b. Along the line of incongruence, creative thinking is higher when participation in out-of-school creative activities exceeds participation in in-school creative activities than in the reverse direction (v1 > 0).

Previous studies indicate that creativity is influenced by both students’ openness to experience and the openness of the school environment (Du et al., 2019; Kaufman et al., 2015). Therefore, examining the correspondence between these two forms of openness may provide a better understanding of creative thinking.

- RQ3. How is creative thinking related to the correspondence between students’ openness to experience and the school’s culture of openness?

   ○  H3a. Along the line of congruence, creative thinking increases as student openness and school openness increase together (u1 > 0).
   ○  H3b. Along the line of incongruence, creative thinking is higher when student openness exceeds school openness than in the reverse direction (v1 > 0).

## 3 Method

This study employed a cross-sectional and relational survey design based on data from students and schools participating in the PISA Creative Thinking Assessment. The study examined how congruence and incongruence between factors supporting creative thinking in school, family and individual contexts predict creativity.

### 3.1 Population and sample

The population of this study consists of 499,843 students from 63 countries and economies that participated in the PISA 2022 Creative Thinking Assessment (OECD, 2024). The data were obtained from the OECD PISA 2022 public-use database, including the student, school, and cognitive assessment files (OECD, 2024), merged on country and student identifiers. Because the three congruence models involve different predictor pairs, listwise deletion was applied separately within each model on that model’s own variable set (the two focal predictors, ESCS, the 10 plausible values of the criterion, and the 10 plausible values of each achievement control and the final and replicate weights). Consequently, the three models rest on partly different analytic samples, which are reported separately. The model examining congruence between the creative family environment (CREATFAM) and the creative school learning environment (CREATSCH) was estimated with 387,147 students from 61 countries and 16,667 schools. The model examining congruence between in-school creative activity engagement (CREATAS) and out-of-school creative activity engagement (CREATOOS) was estimated with 326,648 students from 62 countries and 16,753 schools. Finally, the model examining congruence between the school’s culture of openness to creativity (OPENCUL) and students’ creative and intellectual openness (CREATOP) was estimated with 365,361 students from 62 countries and 15,252 schools. Missing data were limited to the questionnaire indices and ranged from 8.9 to 30.1%. Plausible values, achievement controls, and all 81 weight variables were complete. Costa Rica was excluded from all models because ESCS was not available, while Israel was excluded from the first model because CREATFAM was not available. Before analysis, the two predictors in each model were centered on their weighted grand means, and the polynomial terms were created from the centered variables. No additional transformation or recoding was applied. The preprocessed data files are provided as Supplementary materials.

### 3.2 Data source and variables

The data for this study were compiled from students’ cognitive test results, background questionnaires and school questionnaires. The variables and their item sources were identified using the PISA 2022 questionnaires, codebooks, and Technical Report (OECD, 2024). CREATFAM was based on six items in ST336, CREATSCH on six items in ST335, CREATAS on eight items in ST337, CREATOOS on eight items in ST338, CREATOP on 10 items in ST340, and OPENCUL on nine items in SC208. CREATFAM, CREATSCH, CREATOP, and OPENCUL used four-point agreement response scales, whereas CREATAS and CREATOOS used frequency response scales. These variables were used as the IRT-scaled indices provided in the PISA database, with higher scores indicating higher levels of the relevant construct.

The dependent variable of the study, cognitive creative thinking, was measured with 10 plausible values (PV1-10CRTH_NC). These plausible values are based on the final pool of 32 creative-thinking assessment items and represent estimated performance on the PISA creative-thinking scale (OECD, 2024). It is important to note that the PISA 2022 creative thinking scale is not reported on the conventional PISA proficiency metric (M = 500, SD = 100). It is a 0–60-point scale and in the present analytic samples it had a weighted mean of 29.13 and a standard deviation of 12.94 (see Table 1). Regression coefficients should therefore be interpreted against this metric: a coefficient of 1.00 corresponds to roughly 0.077 standard deviations of creative thinking. Due to the nature of PISA’s data structure, the 10 plausible value variables generated as creative thinking scores were analyzed separately and pooled results were obtained in accordance with Rubin (1987) rules. Mathematics, reading, and science achievement were likewise represented by their 10 PISA plausible values, while socioeconomic status was measured using the PISA index of economic, social and cultural status (ESCS).

**Table 1:** Descriptive statistics and intercorrelations of study variables.

Variable	M	SD	1	2	3	4	5	6	7
Environmental support model									
1. Family environment	0.003	1.029	–						
2. School environment	0.153	1.058	0.493	–					
3. Creative thinking	29.129	12.938	0.117	−0.015	–				
4. Mathematics	448.585	100.875	0.08	−0.037	0.703	–			
5. Science	458.087	104.176	0.089	−0.036	0.712	0.882	–		
6. Reading	446.167	107.564	0.114	−0.032	0.719	0.819	0.829	–	
7. ESCS	−0.302	1.091	0.116	−0.031	0.408	0.447	0.43	0.417	–
Behavioral engagement model									
1. Activity in school	0.208	1.128	–						
2. Activity out of school	0.319	1.158	0.762	–					
3. Creative thinking	28.951	12.995	−0.246	−0.305	–				
4. Mathematics	447.688	101.566	−0.258	−0.326	0.708	–			
5. Science	456.527	104.719	−0.269	−0.334	0.715	0.883	–		
6. Reading	444.305	108.444	−0.293	−0.359	0.723	0.821	0.83	–	
7. ESCS	−0.289	1.091	−0.098	−0.126	0.416	0.454	0.436	0.425	–
Value orientation model									
1. Open culture	0.138	1.058	–						
2. Openness	0.067	1.024	0.059	–					
3. Creative thinking	29.049	12.972	0.038	0.101	–				
4. Mathematics	447.704	101.355	0.016	0.051	0.707	–			
5. Science	456.973	104.694	0.028	0.071	0.715	0.883	–		
6. Reading	445.545	108.286	0.042	0.079	0.722	0.82	0.83	–	
7. ESCS	−0.299	1.092	0.054	0.071	0.411	0.447	0.43	0.419	–

All statistics are computed within each model’s own analytic sample using senate weights (each country weighted to 1,000 cases). Statistics for Creative Thinking, Mathematics, Science and Reading are averaged over the 10 plausible values; correlations are likewise averaged across the 10 within-draw correlation matrices.

Three different congruence pairs were defined in the study.

Environmental Support Congruence: The first variable of this congruence pair is the CREATSCH index, which measures students’ perceptions of how much their formal educational environments (teachers and school structure) nourish creativity. The second variable is the CREATFAM index, which measures students’ perceptions of how much their immediate environment (friends and family) encourages and supports creative thinking.

Behavioral Engagement Congruence: The Creative Activities at School (CREATAS) variable is an action-based index showing how frequently students participate in creative activities offered within the school, such as painting, drawing, art classes, or debate clubs. Creative Activities Outside School (CREATOOS) measures how frequently students allocate time to the same creative activities outside of school hours and boundaries, in their own social lives (courses, hobbies, independent activities).

Value Orientation Congruence: In this model, the first independent variable is the openness culture (OPENCUL) variable, representing the orientation of students at the school toward openness and creativity. This variable was obtained from the school questionnaire answered by school principals and this school-level score was disaggregated into the dataset to represent all participating students in that school. The second independent variable, creativity and intellectual openness (CREATOP), measures students’ beliefs in their own creativity, the intrinsic satisfaction they derive from creative actions and how open and willing they are toward mental/intellectual challenges.

In the estimation of all three models, four variables known to be directly related to creative thinking served as control variables. To control for the effect of academic achievement domains on creativity, plausible values for mathematics (PV1-10MATC), science (PV1-10SCIC) and reading (PV1-10REAC) were included in the models. Additionally, students’ Economic, Social and Cultural Status (ESCS) index was another control variable in the models. Control variables that are plausible values were also analyzed across 10 plausible values like the dependent variables. All six focal predictors are PISA-scaled WLE indices standardized to an OECD mean of 0 and a standard deviation of 1, so that the two members of each congruence pair share a common metric. This satisfies the commensurability condition required for valid response surface analysis (Edwards and Parry, 1993; Shanock et al., 2010). Descriptive statistics (means, standard deviations, ranges) and intercorrelations for all focal and control variables, computed separately within each model’s analytic sample using senate weights, are reported in Table 1.

The decision to control for mathematics, science and reading achievement warrants explicit justification, because all four cognitive measures are PISA plausible values that share general-ability and common-method variance. In the present samples the creative thinking scale correlates 0.71 with mathematics, 0.71 with science and 0.72 with reading (Table 1). Conditioning on these measures therefore removes not only confounding due to general cognitive ability but potentially also part of the substantive pathway through which family and school environments influence creative thinking, namely via academic engagement and developed ability. The controls were retained because the research question concerns the incremental role of contextual congruence over and above measured achievement, which is the more conservative test. The consequence is that the reported congruence coefficients should be read as lower bounds on the total association between context and creative thinking. Models without controls are reported alongside the full models throughout (see the *R*^2^ values beneath Tables 2–4) so that readers can assess both specifications.

**Table 2:** Design-based Wald test (D1) results of the cubic model against generalized polynomial regression models.

Model pair	Model comparison	*n* Plausible	Δ df	D1 (Wald)	df1	df2	p
Family creative environment vs. school creative environment	Additive vs. CUBIC	10	7	19.734*	7	230.861	<0.001
	Interaction vs. CUBIC	10	6	7.220*	6	195.662	<0.001
Activity at school vs. activity out of school	Additive vs. CUBIC	10	7	31.561*	7	233.438	<0.001
	Interaction vs. CUBIC	10	6	35.801*	6	197.249	<0.001
Openness culture at school vs. openness of students	Additive vs. CUBIC	10	7	5.714*	7	226.516	<0.001
	Interaction vs. CUBIC	10	6	6.905*	6	188.941	<0.001

^*^p < 0.05. The reported D1 statistic is a design-based Wald test of the joint null hypothesis that the coefficients omitted from the restricted model are zero, computed from the pooled coefficient vector and its BRR/Rubin covariance matrix across the 10 plausible values.

**Table 3:** Prediction of creative thinking based on environmental support congruence: cubic polynomial regression coefficients.

Role	Coefficient	Estimate	SE	*Z*	*p*
	Intercept (b₀)	−10.073	15.488	−0.650	0.515
IV	Family (b1)	0.674***	0.056	12.001	<0.001
	Family2 (b3)	−0.053***	0.015	−3.398	<0.001
	Family3 (b6)	−0.024	0.013	−1.854	0.064
	School (b2)	−0.063	0.052	−1.204	0.228
	School2 (b5)	−0.056***	0.017	−3.351	<0.001
	School3 (b9)	0.021*	0.00984	2.141	0.032
	Family × School (b4)	−0.057**	0.022	−2.612	0.009
	Family^2^ × School (b7)	0.033*	0.016	2.038	0.042
	Family × School^2^ (b8)	−0.041***	0.012	−3.423	<0.001
Control variable	Mathematics	0.030***	0.00100	29.580	<0.001
	Reading	0.034***	0.00058	58.509	<0.001
	Science	0.025***	0.00092	26.586	<0.001
	ESCS	0.710***	0.031	22.799	<0.001
LOC	u1	0.611***	0.063	9.638	<0.001
	u2	−0.166***	0.013	−12.464	<0.001
	u3	−0.010	0.014	−0.717	0.474
LOIC	v1	0.737***	0.088	8.371	<0.001
	v2	−0.051	0.041	−1.241	0.214
	v3	−0.119***	0.032	−3.683	<0.001
Variance components	Var(country) τ₀₀	6.897			
	Var(residual) σ^2^	67.442			
	ICC (unconditional)	0.171			
	ICC (conditional)	0.093			

*p < 0.05. **p < 0.01. ***p < 0.001. Level 1 = student level, Level 2 = country level. Model with control variables: *R*^2^ Level 1 = 0.551, *R*^2^ Level 2 = 0.778, total *R*^2^ = 0.590. Model without control variables: *R*^2^ Level 1 = 0.038, *R*^2^ Level 2 = 0.002, total *R*^2^ = 0.030.

**Table 4:** Prediction of creative thinking based on behavioral engagement congruence: cubic polynomial regression coefficients.

Role	Coefficient	Estimate	SE	*Z*	*p*
	Intercept (b₀)	−9.398	9.635	−0.975	0.329
IV	Activity_Out (b1)	−0.397***	0.057	−7.004	<0.001
	Activity_Out2 (b3)	−0.356***	0.056	−6.420	<0.001
	Activity_Out3 (b6)	0.101***	0.017	5.767	<0.001
	Activity_Shc. (b2)	−0.137*	0.070	−1.970	0.049
	Activity_Shc.2 (b5)	−0.167***	0.039	−4.270	<0.001
	Activity_Shc.3 (b9)	0.047***	0.012	4.017	<0.001
	Activity_Out × Activity_Shc. (b4)	−0.132**	0.046	−2.875	0.004
	Activity_Out2 × Activity_Shc. (b7)	0.027	0.018	1.517	0.129
	Activity_Out × Activity_Shc.2 (b8)	−0.00051	0.016	−0.032	0.975
Control variable	Mathematics	0.030***	0.001	28.341	<0.001
	Reading	0.034***	0.001	63.479	<0.001
	Science	0.024***	0.001	25.014	<0.001
	ESCS	0.781***	0.035	22.561	<0.001
LOC	u1	−0.534***	0.048	−11.096	<0.001
	u2	−0.655***	0.051	−12.724	<0.001
	u3	0.174***	0.013	13.584	<0.001
LOIC	v1	−0.259*	0.117	−2.209	0.027
	v2	−0.392***	0.096	−4.061	<0.001
	v3	0.026	0.047	0.548	0.584
Variance components	Var(country) τ₀₀	6.502			
	Var(residual) σ^2^	67.441			
	ICC (unconditional)	0.177			
	ICC (conditional)	0.088			

*p < 0.05. **p < 0.01. ***p < 0.001. Note. Level 1 = student level, Level 2 = country level. Model with control variables: *R*^2^ Level 1 = 0.551, *R*^2^ Level 2 = 0.799, total *R*^2^ = 0.595. Model without control variables: *R*^2^ Level 1 = 0.077, *R*^2^ Level 2 = 0.030, total *R*^2^ = 0.067.

### 3.3 Data analysis

#### 3.3.1 Polynomial regression and RSA model

To test the research hypotheses, a cubic polynomial regression model (CUBIC) was fitted for each congruence pair. Response surface analysis (RSA) is an approach that visualizes in three-dimensional space how combinations of two independent variables predict a dependent variable (Edwards and Parry, 1993). The standard RSA model is written as:


$$Z=b_{0}+b₁X+b₂Y+b₃X^{2}+b₄\mathrm{XY}+b_{5}Y^{2}+e$$

Here, *X* and *Y* represent the two predictor variables forming a congruence pair and *Z* represents the outcome variable (creative thinking score). Humberg et al. (2022) showed that quadratic RSA fails to capture asymmetric and level-dependent congruence effects and extended the model with cubic terms:


$$\begin{array}{l} Z=b_{0}+b₁X+b₂Y+b₃X^{2}+b₄\mathrm{XY}+b_{5}Y^{2}+b_{6}X^{3}\\ +b_{7}X^{2}Y+b_{8}XY^{2}+b_{9}Y^{3}+e \end{array}$$

In this study, three alternative models were estimated for each congruence pair. The first was the additive model (FM3_ADDITIVE), which excludes interaction terms and includes only linear and quadratic terms. The second was the interaction model (FM4_INTER), which excludes cubic terms but includes linear, quadratic and interaction terms. The final model was the cubic model (CUBIC), which includes all linear, quadratic and cubic terms together with their interactions. To determine which model provided the best model-data fit, each restricted model was compared with the cubic model using a design-based Wald test of the joint null hypothesis that the omitted coefficients are zero, computed from the pooled coefficient vector and its BRR/Rubin covariance matrix. A Wald test was used in place of the pooled likelihood-ratio (D3) test of Rubin (1987) because senate weighting and balanced repeated replication yield only a pseudo-likelihood, under which the naive likelihood-ratio statistic does not follow its nominal null distribution (Lumley and Scott, 2014). The results are presented in Table 2. In all three congruence pairs, the cubic model fitted significantly better than both restricted models (*p* < 0.001). Therefore, the results of the CUBIC model were considered in this study.

All models were fitted using PISA student weights. Because students are nested within countries and the pooled international sample cannot be treated as a single homogeneous population, all three models were estimated as two-level random-intercept models with country as the second level. The Level-1 weight was the final student weight rescaled to a within-country mean of one (cluster-mean scaling), and the Level-2 weight was set to one for every country, so that each education system contributes equally to the between-country component. Because the analytic samples differ across models, the rescaling divisor was computed separately within each model. The same country-specific divisor was applied to all 80 replicate weights, preserving the between-replicate variation on which the variance estimator depends. For the descriptive statistics and correlations reported in Table 1, senate weights were used instead: the final student weight W_FSTUWT was multiplied by a country-specific factor f_c = 1,000/Σ_c W_FSTUWT, so that every retained country contributes exactly 1,000 weighted cases. Within any given country the two schemes are proportional, so they imply the same relative weighting of students and differ only in how countries are scaled against one another. Standard errors account for PISA’s stratified and clustered design through balanced repeated replication (BRR) with Fay’s adjustment of k = 0.5 across the 80 replicate weights (variance factor 1/[80(1–0.5)^2^] = 0.05). Plausible values were matched by draw index in line with the international operational standard: the *i*-th plausible value of the criterion was always analyzed together with the *i*-th plausible value of each control (mathematics, science, reading; *i* = 1,…,10). This yields 10 parallel models rather than a 10 × 10 × 10 crossing, and preserves the joint posterior of the latent proficiencies generated by the population model (OECD, 2024). Each of the 10 models was estimated under all 81 weights (1 full-sample + 80 replicates); point estimates and standard errors were then combined with Rubin's (1987) rules for the imputation component and BRR for the sampling component. All analyses were conducted in R 4.6.1 using the BIFIEsurvey package (version 3.8.0) (Robitzsch and Oberwimmer, 2026).

#### 3.3.2 Response surface properties

Response surface analysis (RSA), the analytical method used in this study, is an advanced statistical approach for examining how congruence or incongruence between two independent variables predicts an outcome variable (Edwards and Parry, 1993; Schönbrodt, 2016; Shanock et al., 2010). In the RSA method, two basic surface features are interpreted. For this comparison to be valid, the two variables must be measured on the same metric. That is, the commensurability condition must be satisfied (Edwards and Parry, 1993; Shanock et al., 2010).

Line of Congruence (LOC): Defines the condition *X* = *Y* and encompasses all combinations where the two variables are equal (Edwards and Parry, 1993). When X and Y are set equal along the LOC, the response surface becomes a cubic function of a single variable. The resulting parameters represent the linear, quadratic, and cubic components of this function, respectively. In the cubic model, the LOC is therefore described by u₁ = b₁ + b₂, u₂ = b₃ + b₄ + b₅ and u₃ = b₆ + b₇ + b₈ + b₉ (Humberg et al., 2022). A positive u₁ indicates that increases in both independent variables together are associated with higher creative thinking. The quadratic parameter u₂ describes the curvature of this line: a negative value indicates a concave, inverted U-shaped pattern in which the benefit of additional support diminishes at high levels, whereas a positive value indicates accelerating returns. The cubic parameter u_3_ captures additional higher-order curvature along the LOC, allowing the curvature of the congruence line to vary across levels.

Line of Incongruence (LOIC): Defines the condition *Y* = −*X*, along which the two predictors deviate from each other in opposite directions, with the magnitude of the discrepancy increasing with distance from the point of perfect congruence at the origin (Edwards and Parry, 1993). Substituting this condition into the fitted surface yields a second cubic polynomial, whose coefficients are the three parameters below. In the cubic model, the shape of the surface above this line is described by three parameters. The linear parameter v_1_ = b₁ − b₂ is the slope along the LOIC and indexes the direction of incongruence. A positive value indicates that combinations in which the first predictor exceeds the second predictor are associated with higher creative thinking than the reverse combinations. This is defined as an asymmetric incongruence effect. Because v_1_ and v_3_ change sign when the two predictors are listed in the reverse order, the ordering used throughout is that given in each table heading. The quadratic parameter v_2_ = b₃ − b₄ + b₅ describes the curvature of the surface along the LOIC. A significantly negative v₂ indicates that increasing incongruence in either direction is associated with lower outcome levels, producing a ridge-like pattern along the LOC. The cubic parameter v_3_ = b₆ − b₇ + b₈ − b₉ captures additional higher-order curvature along the LOIC.

Each surface parameter was calculated as a weighted sum of the polynomial coefficients. Standard errors were calculated using the pooled covariance matrix, taking into account both the variances and covariances of the coefficients. This pooled matrix incorporates both BRR sampling variance and variability across the 10 plausible values. Because each parameter is a fixed weighted sum of the coefficients whether those coefficients belong to linear, quadratic or cubic terms this calculation is exact rather than an approximation. Each surface parameter was tested against zero using a two-sided *z* test.

Interpretation of the response surface. The LOC and LOIC parameters should be interpreted together with the corresponding three-dimensional response surface plots. Although the parameters quantify the linear, quadratic, and cubic components of the surface along the two focal lines, the graphical representation provides important information about the overall shape, direction, and magnitude of the response surface.

Predictors were grand-mean centered on their senate-weighted means within each model’s analytic sample before the higher-order terms were formed. Because the control variables (mathematics, science, reading and ESCS) enter the model only as linear terms, their powers and their interactions with X and Y are not included. Therefore, they were not centered. The three achievement controls were divided by 100 during estimation for numerical conditioning and are reported on the original per-point scale; ESCS was left on its published metric.

In the value-orientation model one variable (OPENCUL) is a school-questionnaire construct that has been disaggregated to the student level. Thus, all students within a given school share an identical value. Because the balanced repeated replication weights used resample at the level of the primary sampling unit (schools), they correctly reflect that the effective information content of OPENCUL is bounded by the number of schools rather than the number of students. Therefore, the resulting standard errors are design-consistent rather than treating these within-school-identical values as independent observations. This is the property that a single-level regression with naive (non-design-based) standard errors would lack.

## 4 Results

### 4.1 Findings regarding environmental support congruence

The prediction of creative thinking by family and school creative environment support was examined using a two-level cubic response surface model. The obtained coefficients are presented in Table 3 and the response surface generated from these coefficients is presented in Figure 1. The model including control variables (mathematics, science, reading and ESCS) explained 55.1% of the within-country variance and 77.8% of the between-country variance in creative thinking (*R*^2^Level 1 = 0.551, *R*^2^Level 2 = 0.778, *R*^2^Total = 0.590). When the control variables were removed, the response surface terms alone explained 3.8% of the within-country variance but only 0.2% of the between-country variance (*R*^2^Total = 0.030)Thus, the polynomial congruence terms accounted for a relatively modest proportion of the variance compared with the control variables, with much of the model’s explanatory power associated with academic achievement and socioeconomic status. When the difference between the two levels is examined, the congruence terms are found to operate mainly within countries and contribute very little to distinguishing education systems from one another.

**Figure 1:**
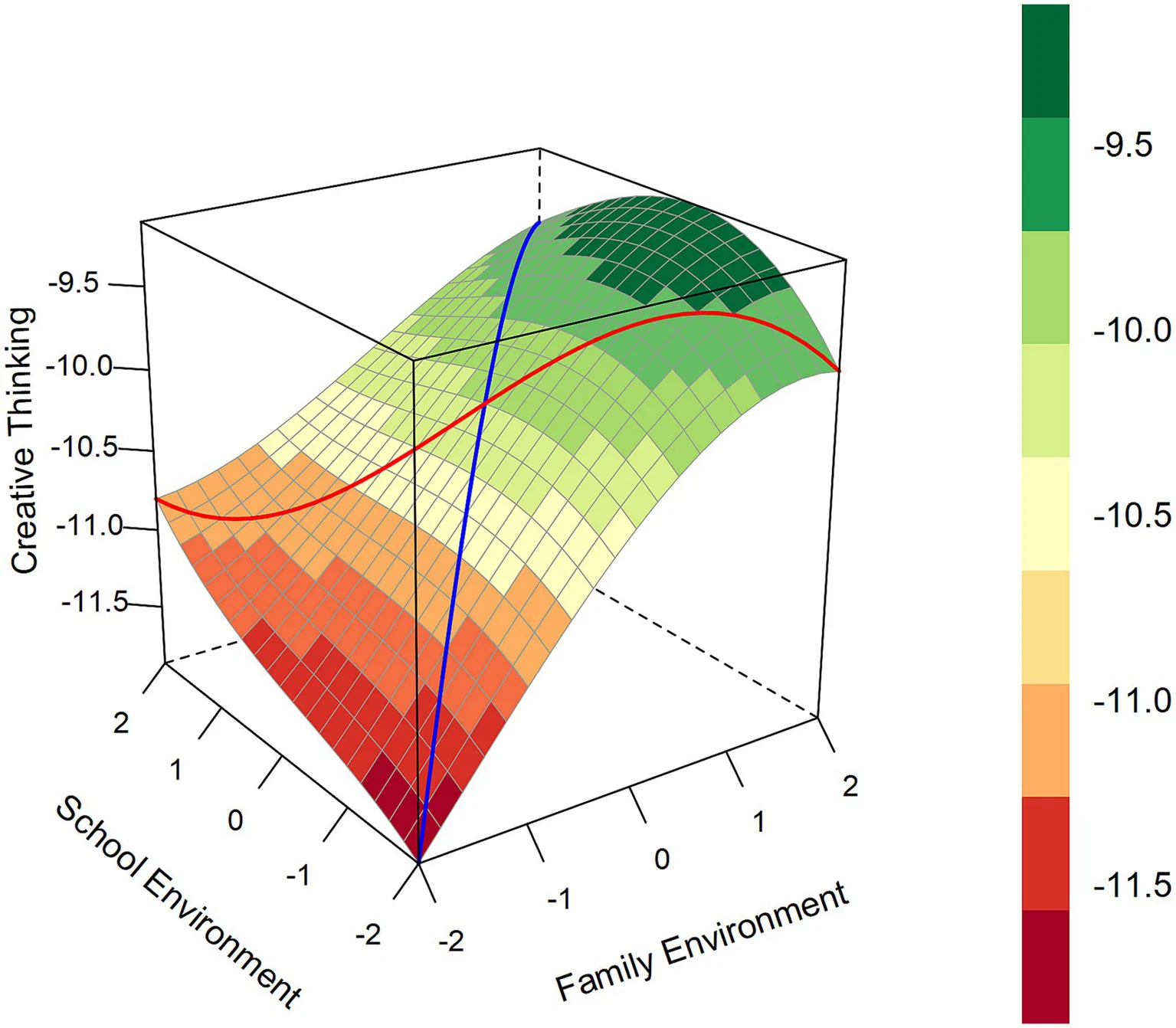
Three-dimensional response surface of family–school environmental support congruence on creative thinking.

When the linear effects of the model were examined, the family creative environment was found to significantly predict the dependent variable (b_1_ = 0.674, *p* < 0.001). The quadratic term of the family creative environment was also negative and significant (b_3_ = −0.053, *p* < 0.001). Although this coefficient indicates a negative quadratic component for the family predictor, the curvature of the overall response surface should be interpreted using the corresponding LOC and LOIC parameters. The cubic coefficient for family creative environment was not significant (b_6_ = −0.024, *p* = 0.064).

For the creative school environment, the linear coefficient was not significant (b_2_ = −0.063, *p* = 0.228), but both higher-order terms were. The quadratic coefficient was negative (b_5_ = −0.056, *p* < 0.001) and the cubic coefficient positive (b_9_ = 0.021, *p* = 0.032). These results indicate that the association between school environment and creative thinking is not adequately described by a linear effect. All three cross-product terms were statistically significant. The linear × linear interaction was negative (b_4_ = −0.057, *p* = 0.009), as was the family × school^2^ term (b_8_ = −0.041, *p* < 0.001), whereas the family^2^ × school term was positive (b_7_ = 0.033, *p* = 0.042). The two environments therefore do not contribute independently to creative thinking: the association between one and the outcome depends on the level of the other, and that dependence is itself non-linear. Such patterns are evaluated through the derived LOC and LOIC parameters, which capture the combined effects of the polynomial terms along the congruence and incongruence lines.

When evaluated in terms of the model response surface parameters, increases in both predictor variables together along the congruence line were associated with a significant positive linear effect on the outcome variable (u_1_ = 0.611, *p* < 0.001), whereas the cubic component along the LOC was not significant (u_3_ = −0.010, *p* = 0.474). The quadratic curvature along the LOC was statistically significant and negative (u_2_ = −0.166, *p* < 0.001). This result indicates that higher congruent levels of family and school creative-environment support are associated with higher creative-thinking scores, while the negative quadratic curvature indicates that this positive association does not remain linear across the full range of the predictors, producing a concave pattern along the LOC.

On the line of incongruence (LOIC), a significant positive linear effect (v_1_ = 0.737, *p* < 0.001) and a significant negative cubic effect (v_3_ = −0.119, *p* < 0.001) were obtained. The quadratic component was not significant (v2 = −0.051, *p* = 0.214). These findings indicate that the discrepancy between family and school creativity environments exhibits a directionally asymmetric and nonlinear relationship with creative thinking. The positive v_1_ indicates that the direction of incongruence matters, with configurations in which family creative environment exceeds school creative environment associated with higher creative-thinking scores than the reverse configuration. The non-significant v_2_ provides no evidence that the two directions of mismatch differ in curvature. The significant negative v_3_, however, indicates that the advantage associated with a family-favoring discrepancy does not accumulate without limit. Evaluating the fitted incongruence function, its slope remains positive across most of the observed range but turns negative beyond approximately 1.3 index points, so that the largest family-favoring discrepancies are associated with lower creative-thinking scores than moderate ones. Because cubic terms are most weakly identified at the edges of the predictor range, this turning point should be read as a description of the fitted surface rather than as a precisely located threshold. The response surface graph (Figure 1) shows the two environments do not contribute symmetrically: the surface rises steeply along the family axis but is comparatively flat along the school axis. When family environment characteristics supporting creativity are high, creative thinking scores remain relatively high despite deficiencies in the school environment. In contrast, high school support is not associated with correspondingly high scores when family support is low. This asymmetry is consistent with the positive slope along the LOIC (v_1_ = 0.737, *p* < 0.001).

### 4.2 Findings regarding behavioral engagement congruence

The polynomial regression results examining the congruence of participation behavior in in-school and out-of-school creative activities are presented in Table 4 and the response surface graph obtained from these results is presented in Figure 2. The model including control variables explained 55.1% of the within-country variance and 79.9% of the between-country variance (R^2^Level 1 = 0.551, R^2^Level 2 = 0.799, R^2^Total = 0.595). When the control variables were removed, the response surface terms alone explained 7.7% of the within-country variance and 3.0% of the between-country variance (R^2^Total = 0.067).

**Figure 2:**
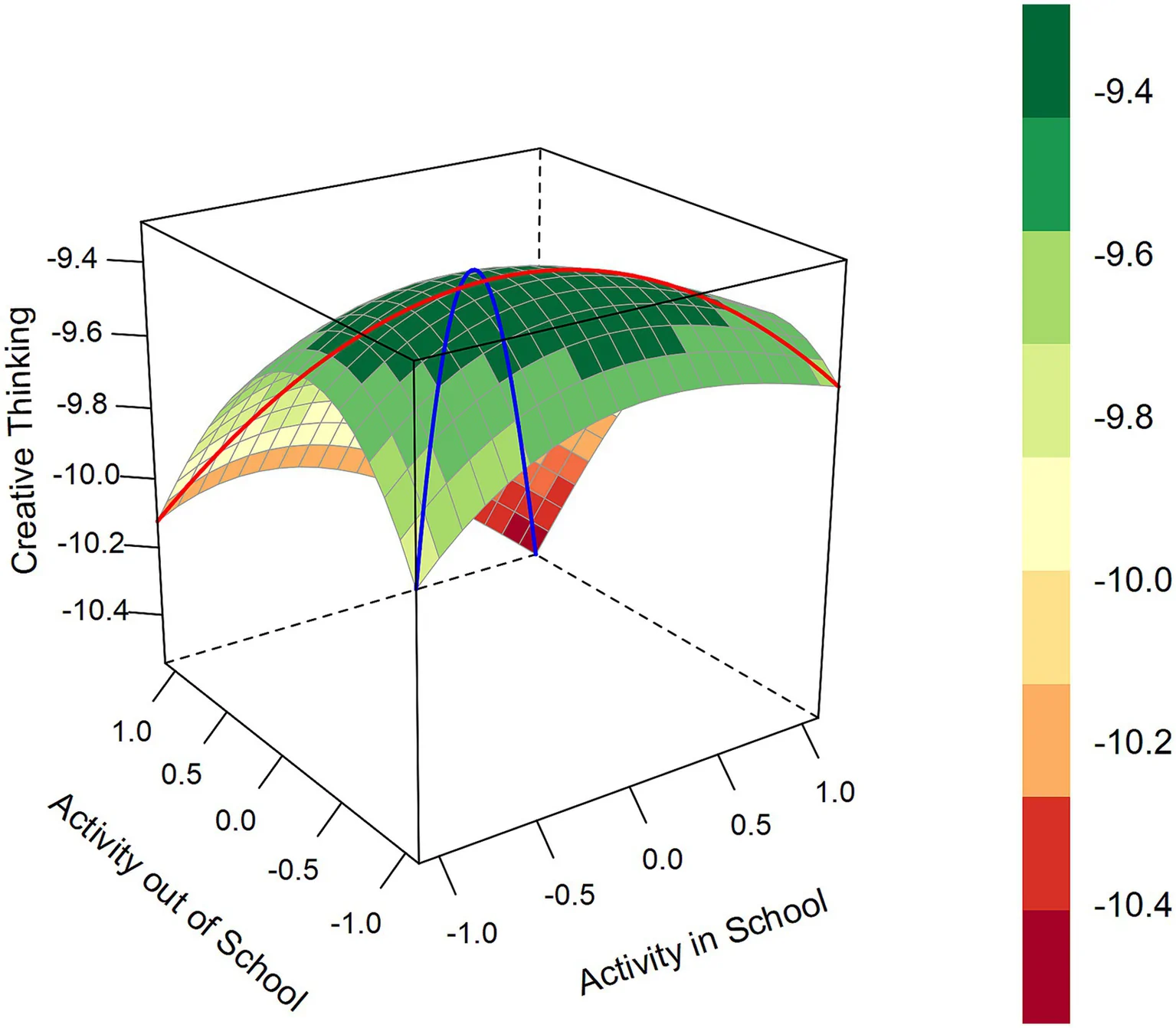
Three-dimensional response surface of congruence between in-school and out-of-school activity engagement on creative thinking.

For out-of-school creative activity participation, the linear coefficient was negative and statistically significant (b_1_ = −0.397, *p* < 0.001), the quadratic coefficient was also negative and significant (b_3_ = −0.356, *p* < 0.001), and the cubic coefficient was positive and significant (b_6_ = 0.101, *p* < 0.001). For in-school creative activity participation, the linear coefficient was negative and statistically significant (b_2_ = −0.137, *p* = 0.049), the quadratic coefficient was negative and significant (b_5_ = −0.167, *p* < 0.001), whereas the cubic coefficient was positive and significant (b_9_ = 0.047, *p* < 0.001). This indicates that, both activity domains therefore show the same qualitative pattern: a negative linear association with negative curvature and a positive third-order component. The linear × linear cross-product term was negative and statistically significant (b_4_ = −0.132, *p* = 0.004), indicating that the two activity domains do not contribute independently. However, the higher-order cross-product terms did not reach significance (b_7_ = 0.027, *p* = 0.129; b_8_ = −0.001, *p* = 0.975).

When the response surface was evaluated along the line of congruence (LOC), the linear surface parameter was negative and statistically significant (u_1_ = −0.534, *p* < 0.001). This indicates that, when participation in in-school and out-of-school creative activities increases concurrently at equal levels, higher levels of participation are associated with lower creative-thinking scores. The quadratic LOC parameter was also negative and significant (u_2_ = −0.655, *p* < 0.001), indicating a concave pattern along the LOC. The cubic component was positive and statistically significant (u_3_ = 0.174, *p* < 0.001), indicating additional higher-order curvature along the congruence line.

Along the line of incongruence (LOIC), the linear parameter was negative and statistically significant (v_1_ = −0.259, *p* = 0.027). This indicates directional asymmetry in the effect of incongruence. Profiles in which out-of-school participation is higher than in-school participation are associated with lower creative-thinking scores compared with profiles showing the opposite pattern. The quadratic LOIC parameter was also negative and significant (v_2_ = −0.392, *p* < 0.001), indicating downward curvature along the LOIC. The cubic LOIC parameter was not significant (v3 = 0.026, *p* = 0.584), providing no evidence of higher-order curvature along the line of incongruence.

Since the relationship along the LOC includes a higher-order cubic component and the LOIC pattern is both asymmetric and nonlinear, the overall shape of both surfaces should be interpreted in conjunction with the three-dimensional response surface shown in Figure 2. Along the LOC, the surface peaks near average levels of balanced participation and declines when students participate at uniformly high levels in both activity domains, indicating that uniformly high matched participation does not yield the highest predicted creative thinking scores. Figure 2 further reveals a nonlinear pattern in which the highest predicted levels of creative thinking are observed when both Activity_Shc and Activity_OUT are around moderate levels. Predicted creative thinking tends to decrease as either activity variable moves toward lower or higher levels, and the surface does not indicate a clear asymmetric compensation pattern.

### 4.3 Findings regarding value orientation congruence

The predictive role of participants’ value orientation congruence (Openness of students and Open Culture of Schools) on creative thinking was tested using a two-level cubic response surface model. The polynomial regression coefficients for the model are presented in Table 5 and the three-dimensional response surface derived from these coefficients is presented in Figure 3. The full model including control variables achieved high predictive power, explaining 55.5% of the within-country variance and 78.0% of the between-country variance in creative thinking (R^2^Level 1 = 0.555, R^2^Level 2 = 0.780, R^2^Total = 0.593). the response surface terms alone explained 5.6% of the within-country variance and 1.1% of the between-country variance (R^2^Total = 0.045). This indicate that the unique contribution of the value orientation congruence terms is modest and that most of the explanatory power derives from academic achievement and socioeconomic status. Moreover, as in the other two models, the congruence terms contribute far more to explaining differences among students within a country than to explaining differences between education systems.

**Table 5:** Prediction of creative thinking based on value orientation congruence: cubic polynomial regression coefficients.

Role	Coefficient	Estimate	SE	*Z*	*p*
	Intercept (b₀)	−10.397	26.356	−0.394	0.693
IV	Openness (b1)	0.708***	0.055	12.917	<0.001
	Openness2 (b3)	−0.078***	0.014	−5.444	<0.001
	Openness3 (b6)	−0.021*	0.009	−2.390	0.017
	Open_Culture (b2)	0.222***	0.064	3.461	<0.001
	Open_Culture2 (b5)	−0.005	0.018	−0.267	0.790
	Open_Culture3 (b9)	−0.005	0.010	−0.506	0.613
	Openness × Open_Culture (b4)	−0.028	0.022	−1.280	0.200
	Openness2 × Open_Culture (b7)	0.013	0.011	1.111	0.266
	Openness × Open_Culture2 (b8)	0.009	0.011	0.817	0.414
Control variable	Mathematics	0.030***	0.001	28.910	<0.001
	Reading	0.035***	0.001	49.990	<0.001
	Science	0.024***	0.001	29.360	<0.001
	ESCS	0.691***	0.040	17.464	<0.001
LOC	u1	0.930***	0.092	10.145	<0.001
	u2	−0.110***	0.029	−3.798	<0.001
	u3	−0.004	0.021	−0.212	0.832
LOIC	v1	0.486***	0.076	6.362	<0.001
	v2	−0.055	0.033	−1.644	0.100
	v3	−0.019	0.022	−0.863	0.388
Variance components	Var(country) τ₀₀	6.692			
	Var(residual) σ^2^	67.022			
	ICC (unconditional)	0.168			
	ICC (conditional)	0.091			

*p < 0.05. **p < 0.01. ***p < 0.001. Level 1 = student level, Level 2 = country level. Model with control variables: *R*^2^ Level 1 = 0.555, *R*^2^ Level 2 = 0.780, total *R*^2^ = 0.593. Model without control variables: *R*^2^ Level 1 = 0.056, *R*^2^ Level 2 = 0.011, total *R*^2^ = 0.045.

**Figure 3:**
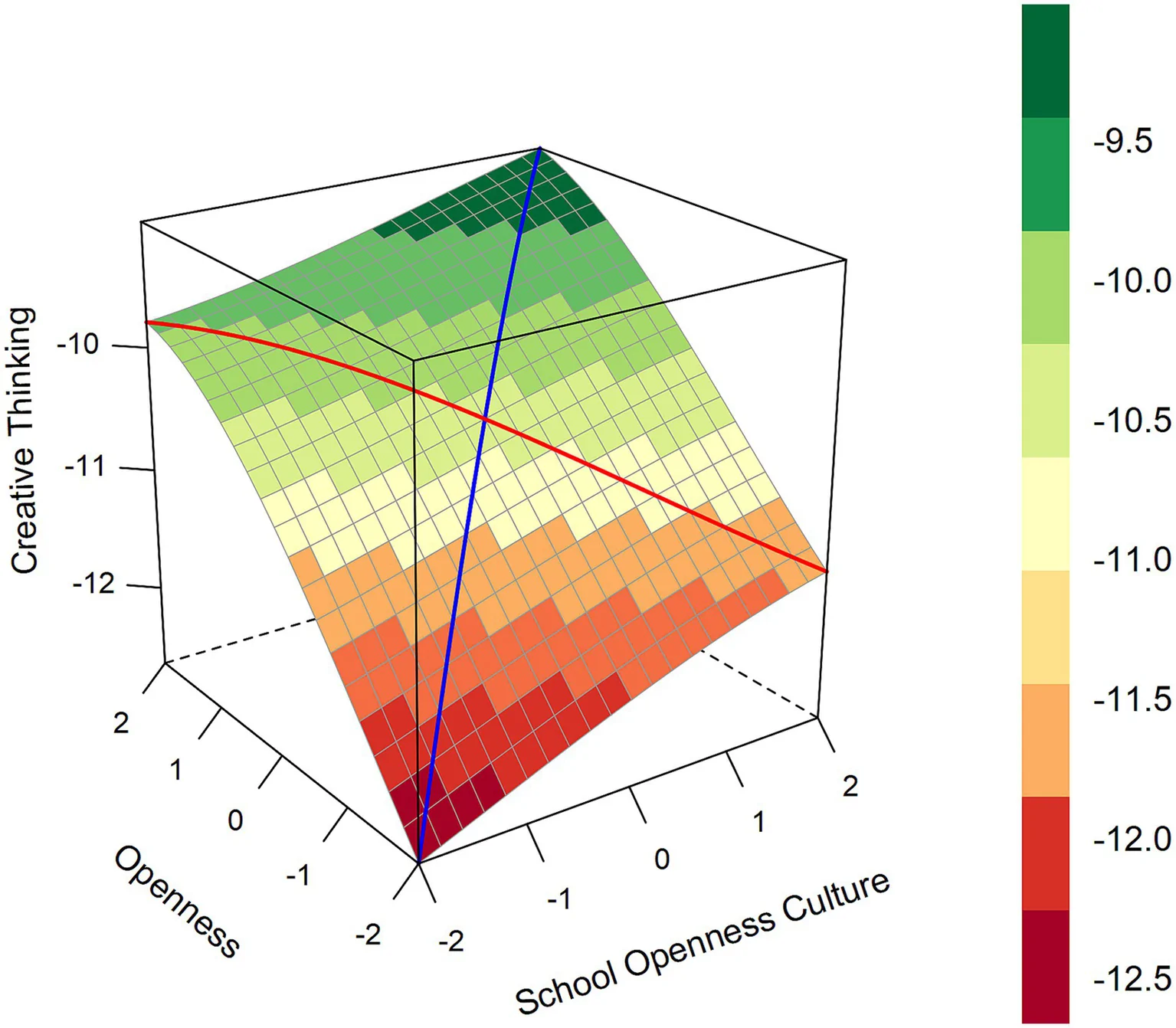
Three-dimensional response surface of congruence between openness culture in schools and openness of students.

For students’ openness to experience, the linear coefficient was positive and statistically significant (b_1_ = 0.708, *p* < 0.001) the quadratic coefficient was negative and significant (b_3_ = −0.078, *p* < 0.001), and the cubic coefficient was also negative and significant (b_6_ = −0.021, *p* = 0.017). For schools’ open culture, the linear coefficient was positive and statistically significant (b_2_ = 0.222, *p* < 0.001), whereas neither the quadratic (b5 = −0.005, *p* = 0.790) nor the cubic (b9 = −0.005, *p* = 0.613) coefficient was statistically significant. The school’s culture of openness therefore enters the surface as an essentially linear effect, while student openness carries significant curvature in addition to a linear component roughly three times its size. None of the three cross-product terms reached statistical significance: the linear × linear term (b_4_ = −0.028, *p* = 0.200), the openness^2^ × open-culture term (b_7_ = 0.013, *p* = 0.266), and the openness × open-culture^2^ term (b8 = 0.009, *p* = 0.414).

When the response surface was evaluated along the line of congruence (LOC), the linear surface parameter was positive and statistically significant (u_1_ = 0.930, *p* < 0.001). This indicates that, when students’ openness to experience and schools’ open culture increase concurrently at equal levels, higher levels of congruent openness are associated with higher creative-thinking scores. The quadratic LOC parameter was negative and significant (u_2_ = −0.110, *p* < 0.001), indicating a concave pattern along the LOC, such that the benefit of jointly increasing openness diminishes at high congruent levels. The cubic component was not statistically significant (u_3_ = −0.004, *p* = 0.832), indicating no additional higher-order curvature along the congruence line.

Along the line of incongruence, the linear parameter was positive and statistically significant (v_1_ = 0.486, *p* < 0.001). This indicates directional asymmetry in the effect of incongruence: configurations in which students’ openness exceeds schools’ open culture are associated with higher creative-thinking scores than the reverse direction of incongruence. Neither the quadratic (v2 = −0.055, *p* = 0.100) nor the cubic (v3 = −0.019, *p* = 0.388) parameter was statistically significant. Therefore, there was no evidence of a nonlinear relationship along the line of incongruence. The relationship between the direction of mismatch and creative thinking can be considered mainly linear. Within the observed range, higher student openness relative to school openness culture was associated with higher creative thinking scores. However, there was no evidence that this relationship became stronger or weaker as the level of mismatch increased. The response surface shown in Figure 3 reveals an asymmetric compensation pattern: high levels of student openness substantially offset the disadvantage associated with low school open culture, whereas high levels of school open culture only partially compensate for low student openness. Consequently, the lowest predicted scores occur where both predictors are low, and among incongruent configurations, profiles in which school open culture exceeds student openness are associated with markedly lower scores than the reverse.

## 5 Discussion

The present study contributes to the creativity literature by examining creative thinking not through isolated predictors, but through the correspondence between different parts of students’ creative ecology. This approach is consistent with the sociocultural perspective of Lubart et al. (2019). According to these researchers, creativity should not be regarded as a property belonging to the individual alone. Rather, it should be understood through the interdependence between the person and the cultural context (Lubart et al., 2019). At the same time, the classroom should also be treated as a specific context, since it is an environment that carries its own constraints and opportunities for supporting creativity, for both teachers and students (Beghetto, 2019). However, the present findings show that correspondence does not operate in the same way for environmental support, behavioral engagement, and value orientation. This divergence is consistent with two lines of research. First, Hennessey (2019) demonstrates that the relation between motivation and creative performance is considerably more complex than the classical intrinsic–extrinsic distinction suggests. She notes that the two types of motivation can, in certain cases, work together rather than against each other. Second, Karwowski et al. (2019) treat creative self-beliefs as a distinct layer and emphasize that what individuals believe about their own creativity shapes how they interpret their environment. Considered together, these two lines indicate that the components of a creative ecology cannot be expected to align in a single, uniform pattern. The present findings support this expectation.

### 5.1 Environmental support congruence

Because the polynomial coefficients of a cubic response surface model are mutually dependent, the congruence hypotheses were interpreted through the surface parameters rather than through the individual b coefficients (Edwards and Parry, 1993; Shanock et al., 2010; Humberg et al., 2019). The findings regarding congruence between family and school creative environment support revealed that the family environment is a strong linear predictor of creative thinking. This result aligns with previous studies emphasizing the central role of family in the creative development process (Amabile, 1983; Runco, 2006; Pugsley and Acar, 2020). Particularly in a large-scale study conducted across eight countries, parental responsiveness showed more consistent and stronger relationships with students’ motivational factors and creative achievement than faculty responsiveness (Zhang et al., 2021). Similarly, constructive parenting style (acceptance and granting autonomy) positively predicted three out of four dimensions of creativity climate in the parent–child relationship (encouraging novelty and diversity, supporting persistence in effort, encouraging daydreaming) (Jankowska and Gralewski, 2022). The cubic term for the family environment was not statistically significant, whereas the negative and significant quadratic term indicated a decelerating trend at higher levels. The school environment showed no significant linear effect, but both its quadratic and its cubic terms were significant. This result indicates that it enters the surface through curvature rather than through a constant association across its range. Evaluated within the response surface as a whole, this pattern reflects that the school creative environment alone, particularly when combined with low family support, remains relatively insufficient. Indeed, the response surface remains comparatively flat along the school axis while rising steeply along the family axis. This means that increases in the school environment alone are not associated with meaningful gains in creative thinking when family support is low. Runco et al. (2017) reported that there are several fundamental incompatibilities between creativity and the school environment: creativity often requires autonomy and independence, while school environments frequently constrain these. Taken together, these findings suggest that the effectiveness of school-based creativity interventions cannot be considered independently of the family environment. One possible reason for this asymmetry is related to the nature of the school context. Beghetto (2019) points out that classrooms offer opportunities for creative expression, but that they also place constraints on it. Creative behavior in this context therefore depends not only on the support that is available, but also on whether students can act on it with a sense of self-determination (Hennessey, 2019). Home environments are less structured in this respect, and they may allow a different form of creative engagement. This may explain why school support alone was not sufficient in the present study. When school support was not accompanied by support at home, its association with creative thinking was weaker. The present findings extend this literature in two ways. First, they show that home support and school support are not interchangeable. Second, they show that the direction of the mismatch between them also matters.

The positive and significant linear parameter on the congruence line shows that the simultaneous increase of family and school creative environments is associated with higher levels of creative thinking. The negative and significant quadratic parameter however, indicates that this strengthening follows a concave rather than an accelerating course. Thus, the return on jointly raising both environments diminishes at high congruent levels. This finding aligns with the core prediction of Person-Environment (P-E) Fit theory: the consistency of the environments in which an individual operates creates is associated with more favorable creative outcomes (Edwards and Parry, 1993; Kristof-Brown and Billsberry, 2013). Livingstone et al. (1997) reported that environmental support (supplies for creativity) has a more dominant effect than individual ability.

The pattern along the line of incongruence is particularly noteworthy. The positive and significant linear parameter indicates that the direction of the mismatch matters: profiles in which family support exceeds school support are associated with higher creative-thinking scores than profiles showing the reverse configuration. The quadratic parameter was not significant, but the cubic parameter was negative and significant, indicating that this advantage does not persist as the gap widens: the slope along the incongruence line flattens and then reverses at large discrepancies. Together these parameters describe a directionally asymmetric, non-monotonic pattern in which a moderate discrepancy in favor of the family is associated with the most favorable outcome, and this may indicate the presence of an optimal tension or constructive incongruence phenomenon. In his study examining the relationship between P-E fit and creative behavior, Choi (2004) revealed that incongruence can yield different outcomes for creativity in certain contexts. Indeed, Csikszentmihalyi (1996) suggested that up to a certain level, environmental inconsistency may enable individuals to think more flexibly and adaptively. However, in cases of large inconsistency, creative thinking markedly declined. This finding is also consistent with the results of the systematic review conducted by van der Zanden et al. (2020).

The asymmetric structure of the response surface graph also offers a theoretically critical finding: the family environment emerges as the dominant compensatory predictor. High family support can partially compensate for deficiencies in the school environment. However, high school support cannot compensate for low family support to the same extent. This asymmetry suggests that basic creative orientations shaped early in life and within the family context are more determinative and enduring compared to later institutional interventions (Simonton, 2000; Runco, 2006).

### 5.2 Behavioral engagement congruence

Findings regarding the congruence of in-school and out-of-school activity participation reveal that the behavioral configuration of participation relates to creative thinking in a markedly different way from environmental support. The negative linear and negative quadratic parameters on the LOC indicate that equal increases in both activity types produce diminishing returns and eventually a declining curve in creative thinking. The positive significance of the cubic component suggests that at the highest participation levels, this negative trend partially reverses, creating a new inflection on the surface.

This finding aligns with the overengagement hypothesis: intensive participation in both domains can create pressure on cognitive capacity, potentially hindering creative productivity (Barron and Harrington, 1981). Indeed, in a meta-analytic review of cognitive domains outside creativity, Fredricks (2012) reported that extracurricular activity participation was positively associated with academic outcomes, but at high intensity levels, diminishing returns emerged due to threshold effects. Similarly, Fredricks and Eccles (2006) found that as the scope and intensity of participation increased, academic adjustment declined after a certain point. Eccles and Barber (2003) stated that participation in extracurricular activities was positively related to educational attainment, but this relationship differed depending on activity type and intensity.

The negative linear parameter on the LOIC is one of the more interpretable parameters in this model, Because v_1_ captures the direction rather than the magnitude of the mismatch. It indicates that profiles in which out-of-school participation exceeds in-school participation are associated with lower creative-thinking scores than profiles showing the opposite configuration. The decline associated with the magnitude of the imbalance is captured instead by the negative and significant quadratic parameter, the cubic parameter was not significant, so there is no evidence of further higher-order curvature along this line. Runco et al. (2017) reported that the correlation between in-school and out-of-school creative activities was only *r* = 0.15. This reveals that congruence between these two seemingly independent contexts in terms of creativity is actually quite important. Runco et al. (2017) also reported that students exhibited significantly more creative activities outside school compared to inside school. In the present data, however, neither domain carried a positive association with creative thinking: both linear coefficients were negative, that for out-of-school participation being approximately three times the larger. What distinguishes the two is therefore the steepness of the decline rather than the direction of the association. Considering the negative directional parameter on the LOIC, the imbalance between in-school and out-of-school contexts may lead students’ creative potential to find expression in only one context and be suppressed in the other.

The negative and significant linear × linear interaction term indicates that the relationship between one activity domain and creative thinking depends on the level of the other. The higher-order interaction terms were not significant, suggesting that the simple product term sufficiently explains this pattern. However, the response surface does not indicate a compensation effect. Predicted creative thinking decreases as participation increases in either domain, with a stronger decline for out-of-school activity. The lowest scores are observed when out-of-school activity is high and in-school activity is low. One possible explanation is that the more structured and guided nature of school activities may influence creative development differently (Barbot et al., 2016). Another possible explanation concerns the way activity was measured in the present study. Frequency of participation indicates how often students take part in an activity, but it does not indicate the quality of that participation. Hennessey (2019) argues that the link between motivation and creative performance cannot be reduced to a simple contrast between intrinsic and extrinsic orientation, and that the two can operate together depending on the conditions of the task. Agnoli et al. (2018) examined creative achievement inside and outside the school environment separately and reported that motivational factors predicted achievement in these two settings. Frequent participation may therefore be associated with creative thinking only when it is accompanied by genuine personal engagement.

This may be relevant for the asymmetry observed here. A high frequency of out-of-school activity does not necessarily reflect sustained creative involvement; it may also reflect routine or passive forms of participation. When such activity is not accompanied by structured school-based experience, it may offer limited opportunity for creative development. This interpretation suggests that behavioral congruence should not be interpreted in the same way as environmental or value congruence, and it should be treated with caution, since the present design did not assess the motivational quality of participation.

### 5.3 Value orientation congruence

Findings regarding value orientation congruence reveal the strongest congruence effect among the three analysis dimensions. The strong positive linear parameter on the LOC demonstrates that simultaneous increases in the student’s openness value and the school’s open culture are associated with stronger creative thinking. The negative and significant quadratic parameter however, shows that this strengthening is concave rather than accelerating; the benefit of jointly increasing openness attenuates at high congruent levels. This finding provides the clearest support obtained here for P-E Fit theory. The absence of significant cross-product terms indicates, however, that the congruence effect is configural rather than synergistic in the multiplicative sense emphasized by Kristof (1996). Karwowski and Lebuda (2016) reported that openness is the strongest predictor of creative self-beliefs. Feist (1998) showed that creative individuals are more open to new experiences and that the largest effect sizes are found in the openness dimension. Spanjol et al. (2015) demonstrated that the quality of value congruence matters, with high–high congruence conditions producing more creativity than low–low congruence conditions. Furthermore, the openness of school culture to creative solutions was found to positively predict teachers’ inquiry-based teaching practices, with teacher self-efficacy serving as a mediator in this relationship (Wei and Chuang, 2023). All these findings are consistent with the strong positive linear effect observed on the LOC.

Along the LOIC, the linear parameter was positive and significant, whereas neither the quadratic nor the cubic parameter reached significance. The relationship along this line is therefore essentially linear, which distinguishes it from the environmental-support model, where a significant cubic component indicated that the advantage of a favorable mismatch eventually reverses. Accordingly, predicted creative thinking increases when the student’s individual openness value exceeds the school’s open culture level, and within the observed range there is no evidence that this advantage either accelerates or attenuates as the discrepancy widens. The response surface indicates a pronounced asymmetry: high student openness substantially offsets the disadvantage of a low school open culture. However, a high-level school open culture only partially compensates for low student openness, and the lowest predicted scores occur where both are low. Because the congruence slope is positive and the largest of the three models, the highest predicted scores occur where openness and open culture are both high, not where student openness alone is high. This suggests that the individual’s internal value structure functions as the more influential of the two components, capable of sustaining creative thinking where the institutional context is comparatively unsupportive. This finding aligns with findings regarding the decisive role of intrinsic motivation in creativity research (Amabile, 1983; Deci and Ryan, 2000). Tan et al. (2019) determined that intrinsic motivation and creative process engagement serially mediate the effect of openness on creativity. Multiple researchers have also reported findings on the role of intrinsic motivation in the relationship between openness and creative thinking (Agnoli et al., 2018; Prabhu et al., 2008).

Taken together, the main contribution of this study is to show that correspondence is not a uniform mechanism across students’ creative ecology. Supportive environments and openness show favorable patterns when both components increase together, whereas activity participation follows a different pattern. By examining both congruence and the direction of incongruence, the study extends previous contextual approaches to creativity and shows that not only the level of individual and environmental factors, but also their configuration, is related to creative thinking.

## 6 Implications

### 6.1 Theoretical implications

The findings of this study offer multi-level theoretical contributions to creativity research. First, the study addresses a gap in the literature by showing that creative outcomes cannot be fully understood by examining environmental and individual factors only as separate predictors. Their correspondence and direction of incongruence also provide theoretically meaningful information. By addressing environmental, behavioral and value congruence dimensions within the same model, the study revealed that these congruence types produce different patterns on creative thinking. This finding suggests that congruence is not a uniform mechanism across different dimensions of students’ creative ecology. The findings also show that perfect congruence does not always result in the highest creativity level. In some cases, limited incongruence in a specific direction supported creative thinking, while excessive incongruence produced negative outcomes. Thus, the study extends existing fit-based explanations by showing that both the direction and the degree of mismatch should be considered when explaining creative thinking.

### 6.2 Practical implications

The research results revealed that school-family collaborations should focus not only on academic achievement but also on developing shared environments that support creativity. Schools may therefore strengthen communication with families about students’ creative opportunities and identify cases where school support is substantially lower than support at home. The research found that maintaining high creative activity participation may not always be functional. Accordingly, it should be remembered that a balanced and sustainable participation pattern may be more functional. Educators should therefore prioritize the quality, autonomy, and meaningfulness of creative activities rather than simply increasing their frequency. In activity design, the emphasis should be placed less on increasing the overall amount of creative activity and more on ensuring that school-based creative activities are not substantially weaker than students’ participation in creative activities outside school. Practices that support students’ curiosity, openness to experience and exploration tendencies can foster creative thinking independently of the school’s overall culture. However, the fact that the highest predicted scores occur when both individual openness and institutional openness are high indicates that the institutional climate should not be overlooked. School leaders and teachers should therefore strengthen practices that support open communication, exploration, risk-taking, and the expression of new ideas.

### 6.3 Methodological implications

Another contribution of the study is at the methodological level. The use of multilevel cubic polynomial regression and response surface analysis provided an opportunity to go beyond difference scores or moderation analyses commonly preferred in congruence research. This approach allowed not only the presence of congruence but also the direction, magnitude and form of incongruence to be examined in detail.

## 7 Limitations and future directions

Some limitations should be considered when evaluating the findings. First, the research is based on cross-sectional data from PISA 2022. Therefore, although the relationships obtained present strong predictive patterns, they do not allow for causal interpretation. Additionally, a significant portion of the variables used consist of self-report measures, which may carry the risk of method bias. Future research using longitudinal designs could more clearly reveal the development of congruence and incongruence effects over time. Similarly, studies conducted in different cultural contexts will provide an opportunity to test the extent to which the patterns observed here are universal.

## 8 Conclusion

This study has revealed that creative thinking is shaped not only by individual characteristics or single environmental factors but also by congruence and incongruence patterns between different contexts. For many years, creativity research has treated variables such as family, school, personality traits, or participation in creative activities as independent predictors. However, the effect of how much these factors overlap or diverge from each other on creative thinking has been largely ignored. This study is among the few to examine the congruence and incongruence dynamics between environmental support (family–school), behavioral participation (in-school–out-of-school activities) and value orientation (student openness–school openness culture) within the same research framework using response surface analysis. In contrast to the assumption implicit in much of the existing literature, it revealed that creative thinking does not always benefit most from perfect congruence. Furthermore, the study showed that in some domains, one component can support creative performance even when limited incongruence is observed. In this respect, the study determined that a ‘more support’ approach alone is insufficient for explaining the development of creativity. It demonstrates that how support resources interact with each other is also critical. Across all three models the linear parameter along the line of incongruence was statistically significant. In every case, the full cubic model also provided a significantly better fit than both the additive model and the model including only the two predictors and their cross-product. Thus, neither a difference-score approach, which ignores the level at which correspondence occurs, nor a simple moderation model, which represents the joint effect only through a product term, would have fully captured these patterns.

Another important contribution of the study provides evidence that different incongruence patterns are associated with different creative thinking outcomes. The findings revealed that the family environment can partly compensate for limitations in the school environment. For activity participation, however, the pattern was different and less clear: neither domain showed a positive association with creative thinking, and the direction of mismatch had only a limited role. Finally, student openness appeared to have a stronger positive role than school openness culture. Overall, these findings suggest that creativity development is shaped by asymmetric relationships across different systems. They are also consistent with ecological and system-based approaches to creativity that emphasize the active role of the individual.

## Data Availability

Publicly available datasets were analyzed in this study. This data can be found at: https://www.oecd.org/en/data/datasets/pisa-2022-database.html.
